# Distance plus attention for binding affinity prediction

**DOI:** 10.1186/s13321-024-00844-x

**Published:** 2024-05-12

**Authors:** Julia Rahman, M. A. Hakim Newton, Mohammed Eunus Ali, Abdul Sattar

**Affiliations:** 1 School of Information and Communication Technology, Griffith University, 170 Kessels Rd, Nathan, 4111 QLD Australia; 2 Institute for Integrated and Intelligent Systems (IIIS), Griffith University, 170 Kessels Rd, Nathan, 4111 QLD Australia; 3 School of Information and Physical Sciences, University of Newcastle, University Dr, Callaghan, 2308 NSW Australia; 4 Department of Computer Science & Engineering, Bangladesh University of Engineering and Technology, Palashi, 1205 Dhaka Bangladesh

**Keywords:** Binding affinity, Distance matrix, Donor-acceptor, Hydrophobicity, $\pi$-Stacking, Deep learning, Attention

## Abstract

Protein-ligand binding affinity plays a pivotal role in drug development, particularly in identifying potential ligands for target disease-related proteins. Accurate affinity predictions can significantly reduce both the time and cost involved in drug development. However, highly precise affinity prediction remains a research challenge. A key to improve affinity prediction is to capture interactions between proteins and ligands effectively. Existing deep-learning-based computational approaches use 3D grids, 4D tensors, molecular graphs, or proximity-based adjacency matrices, which are either resource-intensive or do not directly represent potential interactions. In this paper, we propose atomic-level distance features and attention mechanisms to capture better specific protein-ligand interactions based on donor-acceptor relations, hydrophobicity, and $\pi$-stacking atoms. We argue that distances encompass both short-range direct and long-range indirect interaction effects while attention mechanisms capture levels of interaction effects. On the very well-known CASF-2016 dataset, our proposed method, named Distance plus Attention for Affinity Prediction (DAAP), significantly outperforms existing methods by achieving Correlation Coefficient (R) 0.909, Root Mean Squared Error (RMSE) 0.987, Mean Absolute Error (MAE) 0.745, Standard Deviation (SD) 0.988, and Concordance Index (CI) 0.876. The proposed method also shows substantial improvement, around 2% to 37%, on five other benchmark datasets. The program and data are publicly available on the website https://gitlab.com/mahnewton/daap.

**Scientific Contribution Statement**

This study innovatively introduces distance-based features to predict protein-ligand binding affinity, capitalizing on unique molecular interactions. Furthermore, the incorporation of protein sequence features of specific residues enhances the model’s proficiency in capturing intricate binding patterns. The predictive capabilities are further strengthened through the use of a deep learning architecture with attention mechanisms, and an ensemble approach, averaging the outputs of five models, is implemented to ensure robust and reliable predictions.

## Introduction

Conventional drug discovery, as noted by a recent study [1], is a resource-intensive and time-consuming process that typically lasts for about 10 to 15 years and costs approximately 2.558 billion USD to bring each new drug successfully to the market. Computational approaches can expedite the drug discovery process by identifying drug molecules or *ligands* that have high binding affinities towards disease-related *proteins* and would thus form strong transient bonds to inhibit protein functions [2–4]. In a typical drug development pipeline, a pool of potential ligands is usually given, and the ligands exhibiting strong binding affinities are identified as the most promising drug candidates against a target protein. In essence, protein-ligand binding affinity values serve as a scoring method to narrow the search space for virtual screening [5].

Existing computational methods for protein-ligand binding affinity prediction include both traditional machine learning and deep learning-based approaches. Early methods used Kernel Partial Least Squares [6], Support Vector Regression (SVR) [7], Random Forest (RF) Regression [8], and Gradient Boosting [9]. However, just like various other domains [10–14], drug discovery has also seen significant recent advancements [15–18] from the computational power and extensive datasets used in deep learning. Deep learning models for protein-ligand binding affinity prediction take protein-ligand docked complexes as input and give binding affinity values as output. Moreover, these models use various input features to capture the global characteristics of the proteins and the ligands and their local interactions in the pocket areas where the ligands get docked into the proteins.

Recent deep learning models for protein-ligand binding affinity prediction include DeepDTA [19], Pafnucy [20], $K_\text {DEEP}$ [21], DeepAtom [22], DeepDTAF [23], BAPA [5], SFCNN [24], DLSSAffinity [4] EGNA [25], CAPLA [26] and ResBiGAAT [27]. DeepDTA [19] introduced a Convolutional Neural Network (CNN) model with input features Simplified Molecular Input Line Entry System (SMILES) sequences for ligands and full-length protein sequences. Pafnucy and $K_{DEEP}$ used a 3D-CNN with 4D tensor representations of the protein-ligand complexes as input features. DeepAtom employed a 3D-CNN to automatically extract binding-related atomic interaction patterns from voxelized complex structures. DeepDTAF combined global contextual features and local binding area-related features with dilated convolution to capture multiscale long-range interactions. BAPA introduced a deep neural network model for affinity prediction, featuring descriptor embeddings and an attention mechanism to capture local structural details. SFCNN employed a 3D-CNN with simplified 4D tensor features having only basic atomic type information. DLSSAffinity employed 1D-CNN with pocket-ligand structural pairs as local features and ligand SMILES and protein sequences as global features. EGNA introduced an empirical graph neural network (GNN) that utilizes graphs to represent proteins, ligands, and their interactions in the pocket areas. CAPLA [26] utilized a cross-attention mechanism within a CNN along with sequence-level input features for proteins and ligands and structural features for secondary structural elements. ResBiGAAT [27] integrates a deep Residual Bidirectional Gated Recurrent Unit (Bi-GRU) with two-sided self-attention mechanisms, utilizing both protein and ligand sequence-level features along with their physicochemical properties for efficient prediction of protein-ligand binding affinity.

In this work, we consider the effective capturing of protein-ligand interaction as a key to making further progress in binding affinity prediction. However, as we see from the literature, a sequential feature-based model such as DeepDTA was designed mainly to capture long-range interactions between proteins and ligands, not considering local interactions. CAPLA incorporates cross-attention mechanisms along with sequence-based features to indirectly encompass short-range interactions to some extent. ResBiGAAT employs a residual Bi-GRU architecture and two-sided self-attention mechanisms to capture long-term dependencies between protein and ligand molecules, utilizing SMILES representations, protein sequences, and diverse physicochemical properties for improved binding affinity prediction. On the other hand, structural feature-based models such as Pafnucy, $K_{DEEP}$ and SFCNN use 3D grids, 4D tensors, or molecular graph representations. These features provide valuable insights into the pocket region of the protein-ligand complexes but incur significant computational costs in terms of memory and processing time. Additionally, these features have limitations in capturing long-range indirect interactions among protein-ligand pairs. DLSSAffinity aims to bridge the gap between short- and long-range interactions by considering both sequential and structural features. Moreover, DLSSAffinity uses 4D tensors for Cartesian coordinates and atom-level features to represent interactions between heavy atoms in the pocket areas of the protein-ligand complexes. These representations of interactions are still indirect, considering the importance of protein-ligand interaction in binding affinity. EGNA tried to use graphs and Boolean-valued adjacency matrices to capture protein-ligand interactions to some extent. However, EGNA’s interaction graph considers only edges between each pair of a $C_\beta$ atom in the pocket areas of the protein and a heavy atom in the ligand when their distance is below a threshold of $10\mathring{A}$.

Inspired by the use of distance measures in protein structure prediction [14, 28, 29], in this work, we employ distance-based input features in protein-ligand binding affinity prediction. To be more specific, we use distances between donor-acceptor [30], hydrophobic [31, 32], and $\pi$-stacking [31, 32] atoms as interactions between such atoms play crucial roles in protein-ligand binding. These distance measures between various types of atoms could essentially capture more direct and more precise information about protein-ligand interactions than using sequence-based features or various other features representing the pocket areas of the protein-ligand complexes. Moreover, the distance values could more directly capture both short- and long-range interactions than adjacency-based interaction graphs of EGNA or tensor-based pocket area representations of DLSSAffinity. Besides capturing protein-ligand interactions, we also consider only those protein residues with donor, hydrophobic, and $\pi$-stacking atoms in this work. Considering only these selective residues is also in contrast with all other methods that use all the protein residues. For ligand representation, we use SMILES strings. After concatenating all input features, we use an attention mechanism to effectively weigh the significance of various input features. Lastly, we enhance the predictive performance of our model by adopting an ensembling approach, averaging the outputs of several trained models.

We name our proposed method as Distance plus Attention for Affinity Prediction (DAAP). On the very well-known CASF-2016 dataset, DAAP significantly outperforms existing methods by achieving the Correlation Coefficient (R) 0.909, Root Mean Squared Error (RMSE) 0.987, Mean Absolute Error (MAE) 0.745, Standard Deviation (SD) 0.988, and Concordance Index (CI) 0.876. DAAP also shows substantial improvement, ranging from 2% to 37%, on five other benchmark datasets. The program and data are publicly available on the website https://gitlab.com/mahnewton/daap.

## Results

**Fig. 1:**
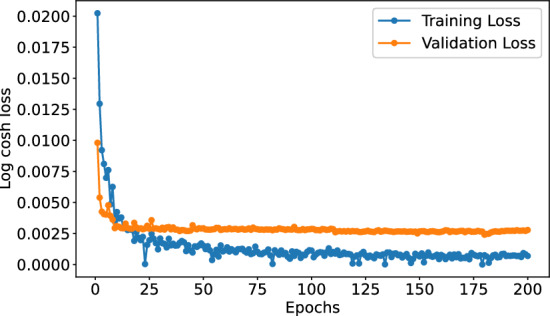
Training and validation loss curve of DAAP

In our study, we first demonstrate the robustness of our deep architecture through five-fold cross-validation. Subsequently, the learning curve, as depicted in Fig. 1, illustrates the dynamics of training and validation loss, providing insights into the stability and reliability of the learning process. Furthermore, we provide a comprehensive performance comparison of our proposed model with current state-of-the-art predictors. We also provide an in-depth analysis of the experimental results. The effectiveness of our proposed features is substantiated through an ablation study and a detailed analysis of input features.

### Five-fold cross-validation

This study employs a five-fold cross-validation approach to evaluate the performance of the proposed model thoroughly, demonstrating the robustness of the deep architecture. Table  1 provides the average performance metrics (R, RMSE, MAE, SD, and CI) along with their corresponding standard deviations derived from the 5-fold cross-validation on the CASF$-$2016.290 test set when the model is trained with PDBbind2016 and PDBbind2020 datasets. This presentation highlights the predictor’s predictive accuracy and reliability, emphasising the proposed model’s effectiveness.

**Table 1:** Average results and Standard Deviation (StdDev) from 5-Fold cross-validation on the CASF$-$2016.290 test set using PDBbind2016 and PDBbind2020 datasets

Train Dataset	Predictors	R$(\uparrow )$	RMSE$(\downarrow )$	MAE$(\downarrow )$	SD$(\downarrow )$	CI$(\uparrow )$
PDBbind2016	Average	0.845	1.196	0.932	1.198	0.825
	StdDev	0.004	0.008	0.006	0.007	0.003
PDBbind2020	Average	0.847	1.183	0.923	1.185	0.827
	StdDev	0.002	0.014	0.020	0.014	0.006

### Average ensemble

Our proposed approach leverages an attention-based deep learning architecture to predict binding affinity. The input feature set comprises distance matrices, sequence-based features for specific protein residues, and SMILES sequences. To enhance the robustness and mitigate the effects of variability and overfitting, we train five models and employ arithmetic averaging for ensembling. Average ensembling is more suitable than max voting ensembling when dealing with real values.

Table 2 shows the results of five models and their averages when all models have the identical setting of their training parameters and the training datasets. We see that the ensemble results are better than the results of the individual models in both the PDBbind2016 and PDBbind2020 training datasets. To check that the proposed approach is robust over the variability in the training datasets, we also train five models but each with a different training subset. These training subsets were obtained by using sampling with replacement. Table 3 shows the results of these five models and their averages.

**Table 2:** Results of five models and their averages when all models were trained using the same training dataset (PDBbind2016 and PDBbind2020) of CASF$-$2016.290

Training Dataset	Predictors	R$(\uparrow )$	RMSE$(\downarrow )$	MAE$(\downarrow )$	SD$(\downarrow )$	CI$(\uparrow )$
PDBbind2016	Model-1	0.860	1.143	0.899	1.144	0.837
	Model-2	0.858	1.152	0.880	1.154	0.835
	Model-3	0.851	1.158	0.895	1.160	0.828
	Model-4	0.858	1.155	0.917	1.157	0.834
	Model-5	0.872	1.094	0.855	1.096	0.843
	Ensemble	**0.886**	**1.067**	**0.836**	**1.069**	**0.853**
PDBbind2020	Model-1	0.878	1.106	0.858	1.108	0.852
	Model-2	0.872	1.120	0.867	1.122	0.845
	Model-3	0.851	1.165	0.898	1.167	0.828
	Model-4	0.855	1.145	0.852	1.147	0.840
	Model-5	0.858	1.133	0.855	1.135	0.839
	Ensemble	**0.909**	**0.987**	**0.745**	**0.988**	**0.876**

Higher R and CI values and lower RMSE, MAE, and SD values denote superior performances. The best-performing values are emboldened

**Table 3:** Results of five models and their averages when each model is trained on distinct subsets of the training dataset (PDBbind2016 and PDBbind2020) for the CASF$-$2016.290

Training Dataset	Predictors	R$(\uparrow )$	RMSE$(\downarrow )$	MAE$(\downarrow )$	SD$(\downarrow )$	CI$(\uparrow )$
PDBbind2016	Model-1	0.872	1.094	0.855	1.096	0.843
	Model-2	0.857	1.168	0.931	1.170	0.831
	Model-3	0.853	1.153	0.906	1.155	0.828
	Model-4	0.862	1.138	0.895	1.140	0.838
	Model-5	0.853	1.183	0.935	1.185	0.829
	Ensemble	**0.888**	**1.061**	**0.827**	**1.063**	**0.853**
PDBbind2020	Model-1	0.878	1.106	0.858	1.108	0.852
	Model-2	0.869	1.128	0.838	1.130	0.847
	Model-3	0.859	1.134	0.840	1.136	0.840
	Model-4	0.852	1.193	0.935	1.195	0.826
	Model-5	0.856	1.227	0.939	1.230	0.828
	Ensemble	**0.908**	**0.999**	**0.732**	**1.001**	**0.874**

Higher R and CI values and lower RMSE, MAE, and SD values denote superior performances. The best-performing values are emboldened

Tables 2 and 3 depict that the ensemble results are better than the results of the individual results in both training sets. It might seem counterintuitive to see the average results are better than all the individual results, but note that these are not simple average of averages. When the ensemble results are compared across Tables 2 and 3, the best results are observed in Table 2 for the PDBbind2020 training set. All evaluation metrics R, RMSE, SD, MAE, and CI display improved performance when using the same training data (Table 2) compared to different varying training data (Table 3) in PDBbind2020 data set. Accordingly, we choose the ensemble with the same training data for PDBbind2020 (Table 2) as our final binding affinity prediction model. Conversely, for PDBbind2016, superior outcomes are obtained from the varied training subsets in Table 3. Henceforth, the best-performing models using PDBbind2016 and PDBbind2020 will be referred to as DAAP16 and DAAP20, respectively, in subsequent discussions.

### Comparison with state-of-the-art methods

In our comparative analysis, we assess the performance of our proposed affinity predictor, DAAP, on the CASF-2016 test set, compared to nine recent state-of-the-art predictors: Pafnucy [20], DeepDTA [19], OnionNet [3], DeepDTAF [23], SFCNN [24] DLSSAffinity [4], EGNA [25], CAPLA [26] and ResBiGAAT [27]. Notably, the most recent predictors have surpassed the performance of the initial four, prompting us to focus our comparison on their reported results. For the latter five predictors, we detail the methodology of obtaining their results as follows:

*DLSSAffinity* We rely on the results available on DLSSAffinity’s GitHub repository, as direct prediction for specific target proteins is not possible due to the unavailability of its trained model.

*SFCNN* Utilizing the provided weights and prediction code from SFCNN, we replicate their results, except for CASF-2013. The ambiguity regarding the inclusion of CASF-2013 data in their training set (sourced from the PDBbind database version 2019) leads us to omit these from our comparison.

*EGNA* We have adopted EGNA’s published results for the CASF-2016 test set with 285 protein-ligand complexes due to differing Uniclust30 database versions for HHM feature construction. We applied EGNA’s code with our HHM features for the other five test sets to ensure a consistent evaluation framework.

*CAPLA* Predictions are made based on the features given in CAPLA’s GitHub, except for the ADS.74 dataset, where we can’t predict results due to the unavailability of feature sets. Their results are the same as their reported results.

*ResBiGAAT* We included ResBiGAAT’s published results in our analysis after encountering discrepancies with their online server using the same SMILES sequences and protein sequences from test PDB files as us. Variations in results, particularly for PDB files with multiple chains, led us to rely on their reported data, as it yielded more consistent and higher accuracies than our attempts.

In Table 4, the first 8 methods, namely Pafnucy, DeepDTA, OnionNet, DeepDTAF, DLSSAffinity, SFCNN, $EGNA^*$ and CAPLA reported on 290 CASF-2016 protein-ligand complexes. To make a fair comparison with these 8 methods, we compared our proposed method DAAP16 and DAAP20 on those 290 protein-ligand complexes. From the data presented in the Table 4, it is clear that our DAAP20 approach outperforms all the 8 predictors, achieving the highest R-value of 0.909, the highest CI value of 0.876, the lowest RMSE of 0.987, the lowest MAE of 0.745, and the lowest SD of 0.988. Specifically, compared to the closest state-of-the-art predictor, CAPLA, our approach demonstrated significant improvements, with approximately 5% improvement in R, 12% in RMSE, 14% in MAE, 11% in SD, and 4% in CI metrics, showcasing its superior predictive capabilities. As 3 of the recent predictors, namely SFCNN, EGNA, and ResBiGAAT, reported their result for the 285 protein-ligand complexes on the CASF-2016 dataset, to make a fair comparison with them as well, we assess our predictor, DAAP, on these 285 proteins as well. From the data presented in Table 4, the results revealed that, across all metrics, DAAP20 outperformed these three predictors on 285 proteins as well. Particularly, compared to the recent predictor ResBiGAAT, our approach demonstrated notable improvements, with around 6% improvement in R, 19% in RMSE, 20% in MAE, and 5% in CI metrics, highlighting its superior predictive capabilities.

**Table 4:** Comparison of our method with other state-of-the-art predictors on the CASF-2016 dataset

Predictors	R$(\uparrow )$	RMSE$(\downarrow )$	MAE$(\downarrow )$	SD$(\downarrow )$	CI$(\uparrow )$
Pafnucy	0.775	1.418	1.129	1.375	0.789
DeepDTA	0.749	1.443	1.148	1.445	0.771
OnionNet	0.816	1.278	0.984	1.280	0.811
DeepDTAF	0.789	1.355	1.073	1.337	0.799
DLSSAffinity	0.790	1.400	1.135	1.404	0.795
SFCNN	0.792	1.328	1.030	1.331	0.798
$EGNA^*$	0.785	1.329	1.105	1.332	0.791
CAPLA	0.843	1.200	0.966	1.202	0.820
DAAP16	0.888	1.061	0.827	1.063	0.853
DAAP20	**0.909**	**0.987**	**0.745**	**0.988**	**0.876**
SFCNN (N = 285)	0.793	1.326	1.028	1.325	0.799
EGNA (N = 285)	0.842	1.258	0.980	–	–
ResBiGAAT (N = 285)	0.853	1.230	0.941	–	0.832
DAAP16 (N = 285)	0.886	1.070	0.837	1.071	0.851
DAAP20 (N = 285)	**0.908**	**0.994**	**0.753**	**0.996**	**0.874**

$EGNA^*$ predicted by using our HHM features

N = 285 indicates that the dataset contains 285 protein-ligand complexes, whereas the rest are evaluated with 290 protein-ligand complexes. Missing values are indicated by “-”. The best values are emboldened

Table 5 presents a comprehensive evaluation of the prediction performance of our proposed DAAP approach on five other well-known test sets CASF$-$2013.87, CASF$-$2013.195 ADS.74, CSAR-HiQ.51 and CSAR-HiQ.36. Across these test sets, our DAAP approaches demonstrate superior predictive performance in protein-ligand binding affinity. On the CASF$-$2013.87 dataset, EGNA surpasses CAPLA with higher R-value and CI-value of 0.752 and 0.767, respectively, while CAPLA records lower RMSE, MAE and SD values of 1.512, 1.197, and 1.521. In contrast, our DAAP20 surpasses both, excelling in all metrics with an R of 0.811, RMSE of 1.324, MAE of 1.043, SD of 1.332, and CI of 0.813, with DAAP16 also delivering robust performance. For the CASF$-$2013.195 test set, a similar trend is observed with our DAAP20 approach outperforming the nearest state-of-the-art predictor by a significant margin of 8%-20% across all evaluation metrics. The DAAP16 approach, not DAAP20, stands out on the ADS.74 dataset by surpassing predictors like Pafnucy, SFCNN and EGNA, showcasing substantial improvements of approximately 12%-37% in various metrics. When evaluating the CSAR-HiQ.51 and CSAR-HiQ.36 datasets against six state-of-the-art predictors, DAAP20 consistently outperforms all, indicating enhancements of 2%-20% and 3%-31%, respectively. Although DAAP16 does not surpass ResBiGAAT in CSAR-HiQ.51, it notably excels in the CSAR-HiQ.36 dataset, outperforming ResBiGAAT in all metrics except MAE. These results underscore the exceptional predictive capabilities of our DAAP approach across diverse datasets and evaluation criteria, consistently surpassing existing state-of-the-art predictors.

**Table 5:** Comparison of our method with other state-of-the-art predictors on additional five test sets

Dataset	Predictors	R$(\uparrow )$	RMSE$(\downarrow )$	MAE$(\downarrow )$	SD$(\downarrow )$	CI$(\uparrow )$
CASF-2013.87	$EGNA^*$	0.752	1.560	1.263	1.569	0.767
	CAPLA	0.716	1.512	1.197	1.521	0.749
	DAAP16	0.801	1.328	1.083	1.336	0.801
	DAAP20	**0.811**	**1.324**	**1.043**	**1.332**	**0.813**
CASF-2013.195	Pafnucy	0.700	1.620	1.320	1.610	–
	DeepDTAF	0.608	2.103	1.737	1.787	0.717
	$EGNA^*$	0.761	1.451	1.200	1.458	0.772
	CAPLA	0.770	1.446	1.154	1.436	0.780
	ResBiGAAT	0.769	1.416	1.126	–	0.783
	DAAP16	0.830	1.311	1.071	1.314	0.816
	DAAP20	**0.866**	**1.203**	**0.924**	**1.206**	**0.846**
ADS.74	Pafnucy	0.515	1.465	1.173	1.453	–
	SFCNN	0.647	1.363	1.051	1.331	0.725
	$EGNA^*$	0.623	1.509	1.211	1.519	0.713
	DAAP16	**0.827**	**0.967**	**0.663**	**0.974**	**0.811**
	DAAP20	0.772	1.109	0.881	1.116	0.783
CSAR-HiQ.51	Pafnucy	0.622	1.944	1.667	1.832	0.698
	DeepDTAF	0.606	2.272	1.862	1.860	0.710
	SFCNN	0.601	1.864	1.481	1.883	0.705
	$EGNA^*$	0.693	1.718	1.329	1.735	0.738
	CAPLA	0.686	1.848	1.550	1.701	0.727
	ResBiGAAT	0.842	1.407	1.047	–	0.840
	DAAP16	0.810	1.417	1.052	1.422	0.810
	DAAP20	**0.868**	**1.204**	**0.845**	**1.216**	**0.850**
CSAR-HiQ.36	Pafnucy	0.566	1.658	1.291	1.649	0.566
	DeepDTAF	0.543	2.765	2.318	1.679	0.670
	SFCNN	0.603	1.693	1.329	1.717	0.709
	$EGNA^*$	0.680	1.457	1.212	1.478	0.707
	CAPLA	0.704	1.454	1.160	1.420	0.760
	ResBiGAAT	0.847	1.005	0.784	–	0.820
	DAAP16	0.868	1.004	0.758	1.058	0.867
	DAAP20	**0.879**	**0.970**	**0.633**	**0.984**	**0.895**

$EGNA^*$ predicted by using our HHM features. Missing values are indicated by “-”. The best values are emboldened

**Fig. 2:**
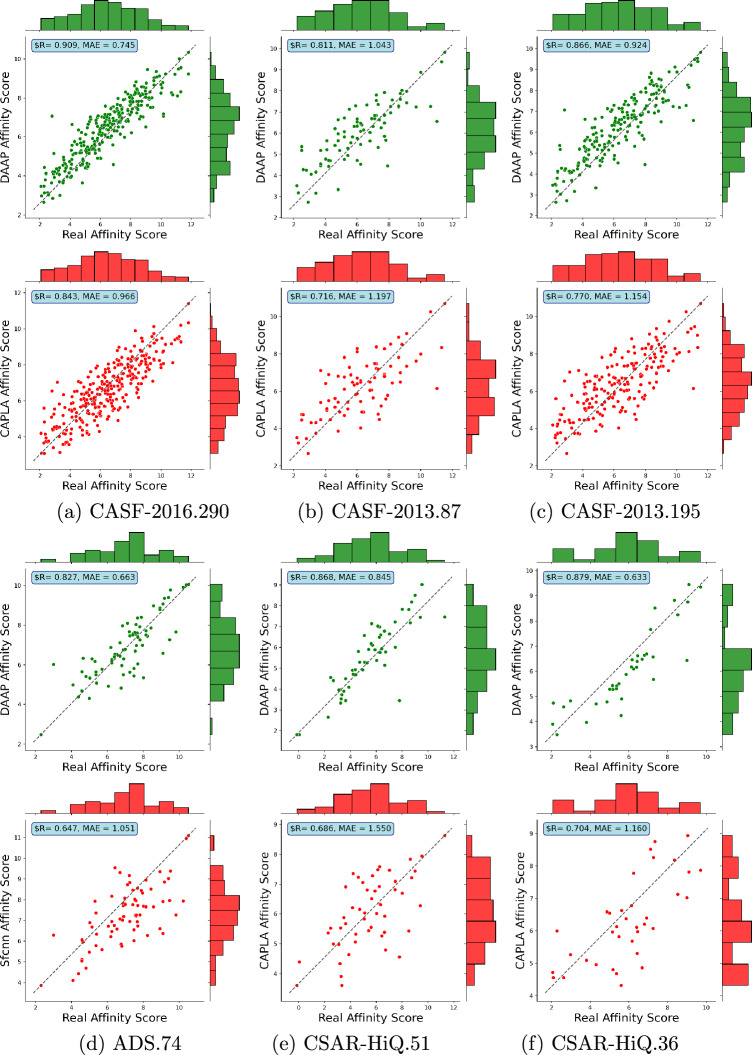
The distributions of real and predicted binding affinity values by our predictor (green) and the closest state-of-the-art predictor (red) across the six test sets

Figure 2 presents the distributions of actual and predicted binding affinities for our best DAAP approach and the closest state-of-the-art predictor. In all six test sets, a clear linear correlation and low mean absolute error (MAE) between predicted and actual binding affinity values can be observed for our DAAP model, demonstrating the strong performance of our model across these test sets. The other predictors show scattering over larger areas. In our analysis, we could not consider ResBiGAAT in the CSAR-HiQ.51 and CSAR-HiQ.36 datasets due to the unavailability of their results.

### Ablation study and explainability

A significant contribution of this work is utilising distance matrix input features to capture critical information about the protein-ligand relationship. Specifically, we employ a concatenation of three distance maps, representing donor-acceptor, hydrophobic, and $\pi$-stacking interactions, as input features, effectively conveying essential protein-ligand bonding details. Following finalising our prediction architecture by incorporating two additional features derived from protein and SMILES sequences, we conduct an in-depth analysis of the impact of various combinations of these distance matrices as features. In the case of protein features, residues are selected based on which distance maps are considered.

Table 6 illustrates the outcomes obtained from experimenting with different combinations of distance maps and selected protein residue and ligand SMILES features on the CASF$-$2016.290 test set. We devise four unique combinations, employing three distinct distance maps for both the PDBbind2016 and PDBbind2020 training datasets. Additionally, we explore a combination that integrates donor-acceptor, hydrophobic, and $\pi$-stacking distance maps with features from all protein residues, denoted as DA + $\pi$S + HP + FP, to evaluate the impact of using all residues versus selected ones.

**Table 6:** Evaluation metrics for various combinations of distance features on the CASF$-$2016.290 test set, including donor-acceptor (DA) distance matrix, $\pi$-stacking ($\pi$S) distance matrix, and hydrophobic (HP) distance matrix between protein and ligand side atoms

Training dataset	Predictors	R$(\uparrow )$	RMSE$(\downarrow )$	MAE$(\downarrow )$	SD$(\downarrow )$	CI$(\uparrow )$
PDBbind2016	DA	0.845	1.189	0.950	1.196	0.825
	DA + $\pi$S	0.846	1.170	0.945	1.173	0.825
	DA + HP	0.851	1.159	0.928	1.162	0.827
	DA + $\pi$S + HP	**0.872**	**1.094**	**0.855**	**1.096**	**0.843**
	DA + $\pi$S + HP + FP	0.837	1.225	0.956	1.227	0.819
PDBbind2020	DA	0.844	1.184	0.939	1.186	0.822
	DA + $\pi$S	0.846	1.174	0.930	1.176	0.825
	DA + HP	0.852	1.168	0.932	1.170	0.826
	DA + $\pi$S + HP	**0.878**	**1.106**	**0.858**	**1.108**	**0.852**
	DA + $\pi$S + HP + FP	0.840	1.204	0.944	1.206	0.821
PDBbind2016	DA + $\pi$S + HP without Attention	0.845	1.206	0.951	1.209	0.822
PDBbind2020	DA + $\pi$S + HP without Attention	0.848	1.199	0.933	1.202	0.826

The best values are emboldened

From the information presented in Table 6, it is evident that utilizing the donor-acceptor (DA) solely distance maps yields the lowest performance across both training sets, particularly when different combinations of distance maps are paired with selective protein residues. However, as expected, the combination of the three distance maps, namely DA, $\pi$S ($\pi$-stacking), and HP (Hydrophobicity), demonstrates superior performance compared to other combinations. Notably, the combination of DA and HP outperforms the other two combinations but falls short of our best-performing feature set. The ensemble of DA, $\pi$S, HP and all protein residues exhibit the least favourable outcomes among the tested combinations. This result aligns with our expectations, as Hydrophobic interactions are the most prevalent in protein-ligand binding, underscoring their significance in feature analysis.

**Fig. 3:**
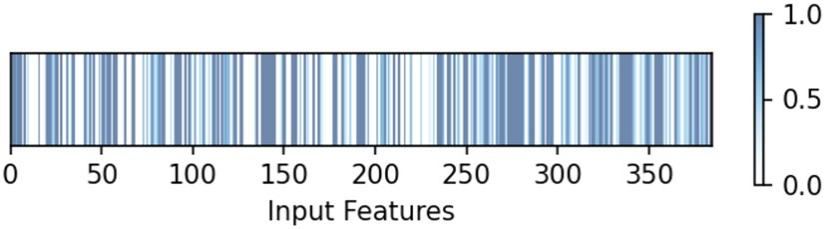
Visualization of attention maps for concatenated features in the 1o0h protein-ligand complex of the CASF$-$2016.290 dataset

Integrating an attention mechanism into our model is crucial in achieving improved results. After consolidating the outputs of three 1D-CNN blocks, we employ attention, each receiving inputs from distance maps, protein sequences, and ligand sequences. The dimension of the feature is 384. As depicted in Fig. 3, the heatmap visualization highlights the differential attention weights assigned to various features, with brighter and darker regions indicating higher weights to certain features, thus improving binding affinity predictions. This process underscores the mechanism’s ability to discern and elevate critical features, showing that not all features are equally important. Further emphasizing the significance of attention, a comparative analysis using the same model architecture without the attention mechanism on the same features-shown in the last row of Table 6 demonstrates its vital role in boosting predictive accuracy. This comparison not only reinforces the value of the attention mechanism in detecting intricate patterns within the feature space but also significantly enhances the model’s predictive capabilities.

### Statistical analysis

In assessing the statistical significance of performance differences between DAAP and its closest competitors, Wilcoxon Signed Ranked Tests at a 95% confidence level were conducted. Comparisons included DAAP against CAPLA for CASF$-$2016.290, CASF$-$2013.87, CASF$-$2013.195, CSAR-HiQ.36, and CSAR-HiQ.51 datasets and between DAAP and SFCNN for the ADS.74 test set. Unfortunately, ResBiGAAT’s results were unavailable for inclusion in the analysis. Table 7 depicts that DAAP demonstrated statistical significance compared to the closest state-of-the-art predictor across various test sets, as indicated by p-values ranging from 0.000 to 0.047. The consistently negative mean Z-values, ranging from $-$14.71 to $-$5.086, suggest a systematic improvement in predictive performance. Moreover, higher mean rankings, ranging from 19.5 to 144.5, further emphasize the overall superiority of DAAP. Notably, the superior performance is observed across diverse datasets, including CASF$-$2016.290, CASF$-$2013.87, CASF$-$2013.195, ADS.74, CSAR-HiQ.51, and CSAR-HiQ.36. These findings underscore the robustness and effectiveness of DAAP in predicting protein-ligand binding affinity.

**Table 7:** Summary of Wilcoxon Signed Ranked and Z Tests on six test sets based

Dataset	p-value	Mean Z-value	Z-Std	Mean ranking	Ranking-Std
CASF-2016.290	0.000	$-$14.71	0.029	144.5	83.715
CASF-2013.87	0.014	$-$7.866	0.054	41.50	24.247
CASF-2013.195	0.015	$-$12.047	0.036	97.00	56.291
ADS.74	0.000	$-$7.374	0.058	36.5	21.36
CSAR-HiQ.51	0.009	$-$6.093	0.069	25.0	14.72
CSAR-HiQ.36	0.040	$-$5.086	0.072	19.5	10.388

### Screening results

In this section, we scrutinize the effectiveness of our predicted affinity scores to accurately differentiate between active binders (actives) and non-binders (decoys) throughout the screening procedure. To this end, we have carefully curated a subset of seven hand-verified targets from the Database of Useful Decoys: Enhanced (DUD-E), accessible via https://dude.docking.org, to serve as our evaluative benchmark. The details about seven targets are given in Table 8. This table underscores the diversity and challenges inherent in the dataset, reflecting a wide range of D/A ratios that present a comprehensive framework for evaluating the discriminatory power of our predicted affinity scores.

**Table 8:** Summary of seven targets from DUD-E dataset

Targets name	No. of actives(A)	No. of decoys(D)	D/A ratio
Adenosine A2a receptor (aa2ar)	844	10899	12.90
Thymidylate synthase (tysy)	311	6883	22.13
MAP kinase-activated protein (mapk2)	206	6244	30.31
Cyclin-dependent kinase (cdk2)	798	28328	35.50
Serine/threonine-protein kinase (akt1)	423	16576	39.19
Tyrosine-protein kinase (src)	831	34959	42.07
Beta-lactamase (ampc)	62	2902	46.80

**Fig. 4:**
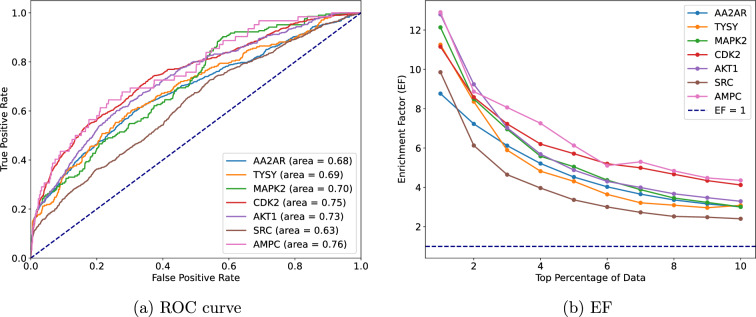
Screening Performance of the Predictive Model: Roc curve (left) and EF (right)

To construct protein-ligand complexes for these targets, we employed AutoDock Vina, configuring the docking grid to a $20\mathring{A} \times 20\mathring{A} \times 20\mathring{A}$ cube centred on the ligand’s position. This setup and 32 consecutive Monte-Carlo sampling iterations identified the optimal pose for each molecule pair. Our evaluation of the screening performance utilizes two pivotal metrics: the Receiver Operating Characteristic (ROC) curve [33] and the Enrichment Factor (EF) [34]. Figure 4 shows the ROC curve and the EF graph for a detailed examination of a predictive model’s efficacy in virtual screening. The ROC curve’s analysis, with AUC values spanning from 0.63 to 0.76 for the seven targets, illustrates our model’s proficient capability in differentiating between actives and decoys. These values, closely approaching the top-left corner of the graph, denote a high true positive rate alongside a low false positive rate, underscoring our model’s efficacy.

Furthermore, the EF graph of Fig. 4 provides a quantitative assessment of the model’s success in prioritizing active compounds within the top fractions of the dataset, notably the top 1% to 10%. Initial EF values ranging from 12.3 to 9.9 for the top 1% underscore our model’s exceptional ability to enrich active compounds beyond random chance significantly. This pronounced enrichment highlights the model’s utility in the early identification of promising candidates. However, the observed gradual decline in EF values with increasing dataset fractions aligns with expectations, reflecting the challenge of sustaining high enrichment levels across broader selections.

## Conclusions

In our protein-ligand binding affinity prediction, we introduce atomic-level distance map features encompassing donor-acceptor, hydrophobic, and $\pi$-stacking interactions, providing deeper insights into interactions for precise predictions, both for short and long-range. We enhance our model further with specific protein sequence features of specific residues and ligand SMILES information. These features are integrated into an attention-based 1D-CNN architecture that is used a number of times for ensemble-based performance enhancement, resulting in superior results compared to existing methods across six benchmark datasets. Remarkably, on the CASF-2016 dataset, our model achieves a Correlation Coefficient (R) of 0.909, Root Mean Squared Error (RMSE) of 0.987, Mean Absolute Error (MAE) of 0.745, Standard Deviation (SD) of 0.988, and Concordance Index (CI) of 0.876, signifying its potential to advance drug discovery binding affinity prediction. The program and data are publicly available on the website https://gitlab.com/mahnewton/daap.

## Methods

We describe the protein-ligand dataset used in our work. We also describe our proposed method in terms of its input features, output representations, and deep learning architectures.

### Protein-ligand datasets

In the domain of protein-ligand binding affinity research, one of the primary sources for training, validation, and test sets is the widely recognized PDBbind database [35]. This database is meticulously curated. It comprises experimentally verified protein-ligand complexes. Each complex encompasses the three-dimensional structures of a protein-ligand pair alongside its corresponding binding affinities expressed as $pK_d$ values. The PDBbind database (http://www.pdbbind.org.cn/) is subdivided into two primary subsets: the *general set* and the *refinement set*. The PDBbind *version 2016* dataset (named PDBbind2016) contains 9221 and 3685 unique protein-ligand complexes, while the PDBbind *version 2020* dataset (named PDBbind2020) includes 14127 and 5316 protein-ligand complexes in the general and refinement sets, respectively.

Similar to the most recent state-of-the-art affinity predictors such as Pafnucy [20], DeepDTAF [23], OnionNet [3], DLSSAffinity [4], LuEtAl [36], EGNA [25] and CAPLA [26], our DAAP16 method is trained using the 9221 + 3685 = 12906 protein-ligand complexes in the general and refinement subsets of the PDBbind dataset *version 2016*. Following the same training-validation set formation approach of the recent predictors such as Pafnucy, OnionNet, DeepDTAF, DLSSAffinity and CAPLA, we put 1000 randomly selected protein-ligand complexes in the validation set and the remaining 11906 distinct protein-ligand pairs in the training set. Another version of DAAP, named DAAP20, was generated using the PDBbind database *version 2020*, which aligns with the training set of ResBiGAAT [27]. To avoid overlap, we filtered out protein-ligand complexes common between the PDBbind2020 training set and the six independent test sets. After this filtering process, 19027 unique protein-ligand complexes were retained for training from the initial pool of 19443 in PDBbind2020.

To ensure a rigorous and impartial assessment of the effectiveness of our proposed approach, we employ six well-established, independent blind test datasets. There is no overlap of protein-ligand complexes between the training sets and these six independent test sets.

*CASF-2016.290* The 290 protein-ligand complexes, commonly referred to as CASF-2016, are selected from the PDBbind *version 2016* core set (http://www.pdbbind.org.cn/casf.php) and have become the gold standard test set for recent affinity predictors such as DLSSAffinity [4], LuEtAl [36], EGNA [25] and CAPLA [26].

*CASF-2013.87 and CASF-2013.195* Similar to the approach taken by DLSSAffinity [4], we carefully curated 87 unique protein-ligand complexes from the CASF-2013 dataset, which originally consists of 195 complexes (http://www.pdbbind.org.cn/casf.php). These 87 complexes were chosen to ensure no overlap with our training set or the CASF-2016 test set. Additionally, we use the entire set of 195 complexes as another test set, named CASF$-$2013.195.

*ADS.74* This test set from SFCNN [24] comprises 74 protein-ligand complexes sourced from the Astex diverse set [37].

*CSAR-HiQ.51 and CSAR-HiQ.36* These two test datasets contain 51 and 36 protein-ligand complexes from the well-known CSAR [38] dataset. Recent affinity predictors such as EGNA [25], CAPLA and ResBiGAAT [26, 27] have employed CSAR as a benchmark dataset. To get our two test datasets, we have followed the procedure of CAPLA and filtered out protein-ligand complexes with duplicate PDB IDs from two distinct CSAR subsets containing 176 and 167 protein-ligand complexes, respectively.

### Input features

Given protein-ligand complexes in the datasets, we extract three distinctive features from proteins, ligands, and protein-ligand binding pockets. We describe these below.

#### Protein representation

We employ three distinct features for encoding protein sequences: one-hot encoding of amino acids, a Hidden Markov model based on multiple sequence alignment features (HHM), and seven physicochemical properties.

In the one-hot encoding scheme for the 20 standard amino acids and non-standard amino acids, each amino acid is represented by a 21-dimensional vector. This vector contains twenty “0 s” and one “1”, where the position of the “1” corresponds to the amino acid index in the protein sequence.

To construct the HHM features, we have run an iterative searching tool named HHblits [39] against the Uniclust30 database (http://wwwuser.gwdg.de/~compbiol/uniclust/2020_06/) as of June 2020. This process allows us to generate HHM sequence profile features for the proteins in our analysis. Each resulting *.hhm* feature file contains 30 columns corresponding to various parameters such as emission frequencies, transition frequencies, and Multiple Sequence Alignment (MSA) diversities for each residue. Like EGNA, for columns 1 to 27, the numbers are transformed into frequencies using the formula $f = 2^{-0.001*p}$, where *f* represents the frequency, and *p* is the pseudo-count. This transformation allows the conversion of these parameters into frequency values. Columns 28 to 30 are normalized using the equation: $f = \frac{0.001*p}{20}$. This normalization process ensures that these columns are appropriately scaled for further analysis and interpretation.

The seven physicochemical properties [14, 29] for each amino acid residue are steric parameter (graph shape index), hydrophobicity, volume, polarisability, isoelectric point, helix probability, and sheet probability. When extracting these three features for protein residues, we focused exclusively on the 20 standard amino acid residues. If a residue is non-standard, we assigned a feature value of 0.0.

In our approach, we initially concatenate all three features sequentially for the entire protein sequence. Subsequently, to enhance the specificity of our model, we employ a filtering strategy where residues lacking donor [40], hydrophobic [31], and $\pi$-stacking [32] atoms within their amino acid side chains are excluded from the analysis. Additionally, to prevent overlap, we select unique residues after identification based on donor, hydrophobic, or $\pi$-stacking atoms for each protein sequence. The rationale behind this filtering is to focus on residues that are actively involved in critical interactions relevant to protein-ligand binding. The resulting feature dimension for each retained protein residue is 58. This feature set includes one-hot encoding of amino acids, a Hidden Markov model based on multiple sequence alignment features (HHM), and seven physicochemical properties. These features are comprehensively summarised in Table 9 for clarity.

**Table 9:** Feature summary for each amino acid residue in proteins, each character of SMILES sequence of ligands, and each atom pair of the binding pocket

	Features	Size	Values	Feature description
Protein	one-hot encoding	21	1 or 0	1 corresponds to the amino acid index, otherwise 0
	HHM	30	real values	various parameters from MSA
	physicochemical properties	7	real values	steric parameter, hydrophobicity, volume, polarisability, isoelectric point, helix probability, sheet probability
Ligand	SMILES encoding	1	integer values	64 unique characters, corresponding to a specific numeric digit ranging from 1 to 64
Pocket	Distance bins	1	integer values	distances between protein and ligand atoms into 41 bins, with each distance corresponding to a numeric digit from 1 to 41

Each protein could have maximum 500 residues and each ligand SMILES sequence could have maximum 150 charaters

Considering the variable numbers of residues that proteins can possess, we have considered a standardized protein sequence length to align with the fixed-size requirements of deep learning algorithms. In our initial experiments exploring various sequence lengths in the datasets, we found that a maximum length of 500 yields better performance in terms of pearson correlation coefficient (R) and mean absolute error (MAE). If the number of selected residues falls below 500, we pad the sequence with zeros; conversely, if it exceeds 500, we truncate it to 500 from the initial position of the sequence. The final dimension of each protein is $500\times 58$.

#### Ligand representation

We use SMILES to represent ligands. SMILES is a widely adopted one-dimensional representation of chemical structures of ligands [41]. To convert ligand properties such as atoms, bonds, and rings from ligand SDF files into SMILES strings, we use the Open Babel chemical tool [42]. The SMILES strings comprise 64 unique characters, each corresponding to a specific numeric digit ranging from 1 to 64. For example, the SMILES string “HC(O=)N” is represented as [12, 42, 1, 48, 40, 31, 14]. In line with our protein representation approach, we set a fixed length of 150 characters for each SMILES string.

**Fig. 5:**
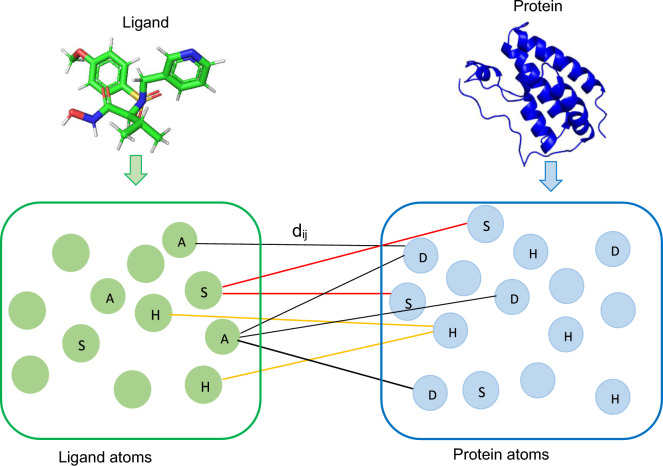
Various distance measures that potentially capture protein-ligand interactions. In the figure, $d_{ij}$ represents the distance between a donor (D), hydrophobic (H), or $\pi$-stacking (S) atom *i* in the protein and the corresponding acceptor (A), hydrophobic (H), or $\pi$-stacking (S) atom *j* in the ligand. Empty circles represent other atom types. Different colour lines represent different types of interactions

#### Binding pocket representation

A binding pocket refers to a cavity located either on the surface or within the interior of a protein. A binding pocket possesses specific characteristics that make it suitable for binding a ligand [43]. Protein residues within the binding pocket region exert a direct influence, while residues outside this binding site can also have a far-reaching impact on affinity prediction. Among various protein-ligand interactions within the binding pocket regions, donor-acceptor atoms [30], hydrophobic contacts [31, 32], and $\pi$-stacking [31, 32] interactions are the most prevalent, and these interactions could significantly contribute to the enhancement of affinity score prediction. The formation of the protein-ligand complexes involves donor atoms from the proteins and acceptor atoms from the ligands. This process is subject to stringent chemical and geometric constraints associated with protein donor groups and ligand acceptors [30]. Hydrophobic interactions stand out as the primary driving force in protein-ligand interactions, while $\pi$-stacking interactions, particularly involving aromatic rings, play a substantial role in protein-ligand interactions [32]. However, there are instances where donor-acceptor interactions alone may not suffice, potentially failing to capture other interactions that do not conform to traditional donor-acceptor patterns. In such scenarios, hydrophobic contacts and $\pi$-stacking interactions become essential as they could provide valuable insights for accurate affinity prediction.

We employ three types of distance matrices in our work shown in Fig. 5 to capture protein-ligand interactions. The first one is the *donor-acceptor distance matrix*, which considers distances between protein donor atoms and acceptor ligand atoms, with data sourced from mol2/SDF files. We ensure that all ligand atoms contribute to the distance matrix construction, even in cases where ligands lack explicit acceptor atoms. Furthermore, we calculate the *hydrophobic distance matrix* by measuring the distance between hydrophobic protein atoms and hydrophobic ligand atoms, ensuring the distance is less than $4.5\mathring{A}$ [31]. Similarly, we compute the $\pi$-*stacking distance matrix* by considering protein and ligand $\pi$-stacking atoms and applying a distance threshold of $4.0\mathring{A}$ [32]. These three types of atoms are selected from the heavy atoms, referring to any atom that is not hydrogen.

We discretize the initially calculated real-valued distance matrices representing the three types of interactions into binned distance matrices. These matrices are constrained within a maximum distance threshold of $20\mathring{A}$. The decision to set a maximum distance threshold of $20\mathring{A}$ for capturing the binding pocket’s spatial context is informed by practices in both affinity prediction and protein structure prediction fields. Notably, methodologies like Pafnucy [20], DLSSAffinity [4], and EGNA [25], as well as advanced protein structure prediction models such as AlphaFold [28] and trRosetta [44], utilize a 20Å range to define interaction spaces or predict structures. This consensus on the 20Å threshold reflects its sufficiency in providing valuable spatial information necessary for accurate modeling. The distance values ranging from $0\mathring{A} - 20\mathring{A}$ are discretized into 40 bins, each with a $0.5\mathring{A}$ interval. Any distance exceeding $20\mathring{A}$ is assigned to the $41^{st}$ bin. In our experimentation, we explored different distance ranges ($20\mathring{A}$, $25\mathring{A}$, $30\mathring{A}$, $35\mathring{A}$, and $40\mathring{A}$) while maintaining a uniform bin interval of $0.5\mathring{A}$. Among these ranges, $20\mathring{A}$ yielded optimal results, and as such, we adopted it for our final analysis. Following this binning process, the original real-valued distances in the matrices are substituted with their corresponding bin numbers. Subsequently, we convert the 2D distance matrix into a 1D feature vector. We concatenate the three 1D vectors representing the three distinct interactions into a single vector to construct the final feature vector. To ensure consistency, the maximum length of the feature vector is set to 1000 for each pocket.

### Output representations

This binding affinity is measured in the dissociation constant ($K_d$). For simplicity in calculations, the actual affinity score $K_d$ is commonly converted into $pK_d$ by taking the negative logarithm of $K_d$.

### Deep learning architectures

**Fig. 6:**
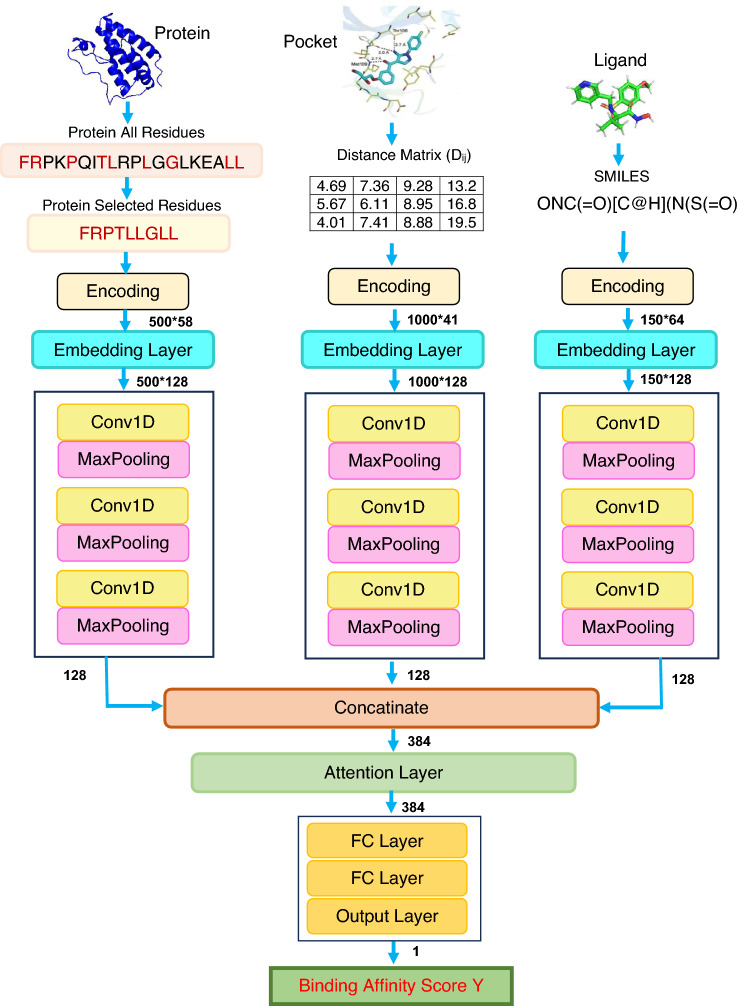
The proposed model architecture

We propose a deep-learning regression model to predict protein-ligand binding affinities, shown in Fig. 6. Our model comprises three integral components: convolutional neural network (CNN), attention mechanism, and fully connected neural network (FCNN). Before feeding to the CNN block, information from three distinct feature sources (proteins, ligands, and interactions) is encoded and subsequently processed through the embedding layer. The embedding layer transforms the inputs into fixed-length vectors of a predefined size (in this case, 128 dimensions), enabling more effective feature representation with reduced dimensionality. During training, our model operates with a batch size of 16 and is optimized using the Adam optimizer and a learning rate set at 0.001. We adopt the log cosh loss function for this work to optimise the model’s performance. The training regimen consists of 200 epochs, with the best model selected based on the validation loss, and a dropout rate of 0.2 is applied. The explored hyperparameter settings are summarised in Table 10. We have explored these settings, and after preliminary experiments, we have selected these values which are emboldened.

**Table 10:** Explored hyperparameters for DAAP Tuning

Parameter	Settings
Number of 1D-CNN filters	16, **32**, **64**, **128**, 256
Filter lengths	2, **4**, 6, **8**, **12**, 16
Number of FC layers	**2**, 3, 4
Node of FC layer	64, **128**, **256**
Dropout rate	0.1, **0**.**2**, 0.3
Loss function	**log cosh**, mse

The final hyperparameters are emboldened

#### Convolutional neural network

Much like DLSSAffinity [4], our model employs three 1D-CNN blocks, each dedicated to processing distinct feature sources: proteins, ligands, and interactions in pockets. Each of these 1D-CNN blocks comprises three convolutional layers paired with three Maxpooling layers. The configuration of the first two 1D-CNN blocks includes 32, 64, and 128 filters, each with corresponding filter lengths of 4, 8, and 12. In contrast, the 1D-CNN block responsible for handling SMILES sequence inputs features filters with 4, 6, and 8 adjusted lengths. Each of the three 1D-CNN blocks in our model generates a 128-dimensional output. Subsequently, before progressing to the next stage, the outputs of these three 1D-CNN blocks are concatenated and condensed into a unified 384-dimensional output.

#### Attention mechanism

In affinity prediction, attention mechanisms serve as crucial components in neural networks, enabling models to allocate varying levels of focus to distinct facets of input data [5]. These mechanisms play a critical role in weighing the significance of different features or entities when assessing their interaction strength. The attention mechanism uses the formula below.

1 $$\begin{aligned} \text {Attention}(Q, K, V) = \text {softmax}\left( \frac{QK^T}{\sqrt{d_k}}\right) V \end{aligned}$$

We use the Scaled Dot-Product Attention [45] mechanism to calculate and apply attention scores to the input data. The attention mechanism calculates query (*Q*), key (*K*), and value (*V*) matrices from the input data. In this context, *Q* is a vector capturing a specific aspect of the input, *K* represents the context or memory of the model with each key associated with a value, and *V* signifies the values linked to the keys. It computes attention scores using the dot product of *Q* and *K* matrices, scaled by the square root of the dimensionality ($d_k$). Subsequently, a softmax function normalises the attention scores. Finally, the output is generated as a weighted summation of the value (V) matrix, guided by the computed attention scores.

Notably, the output of the concatenation layer passes through the attention layer. The input to the attention layer originates from the output of the concatenation layer, preserving the same dimensionality as the input data. This design ensures the retention of crucial structural information throughout the attention mechanism.

#### Fully connected neural network

The output of the attention layer transitions into the subsequent stage within our model architecture, known as the Fully Connected Neural Network (FCNN) block. The FCNN block consists of two fully connected (FC) layers, where the two layers have 256 and 128 nodes respectively. The final stage in our proposed prediction model is the output layer, which follows the last FC layer.

### Evaluation metrics

We comprehensively evaluate our affinity prediction model using five well-established performance metrics. The Pearson Correlation Coefficient (R) [4, 24, 26, 36] measures the linear relationship between predicted and actual values. The Root Mean Square Error (RMSE) [4, 24, 26] and the Mean Absolute Error (MAE) [24, 26] assess prediction accuracy and error dispersion. The Standard Deviation (SD) [4, 24, 26, 36] evaluates prediction consistency, and the Concordance Index (CI) [26, 36] determines the model’s ability to rank protein-ligand complexes accurately. Higher R and CI values and lower RMSE, MAE, and SD values indicate better prediction accuracy. These metrics are collectively very robust measures for comparison of our model’s performance against that of the state-of-the-art techniques in the field of affinity prediction.

2 $$ \begin{gathered}   R = \frac{{\sum\nolimits_{{i = 1}}^{N} {\left( {y_{{{\text{act}}_{i} }}  - \bar{Y}_{{{\text{act}}}} } \right)\left( {y_{{{\text{pred}}_{i} }}  - \bar{Y}_{{{\text{pred}}}} } \right)} }}{{\sqrt {\sum\nolimits_{{i = 1}}^{N} {\left( {y_{{{\text{act}}_{i} }}  - \bar{Y}_{{{\text{act}}}} } \right)^{2} } } \sqrt {\sum\nolimits_{{i = 1}}^{N} {\left( {y_{{{\text{pred}}_{i} }}  - \bar{Y}_{{{\text{pred}}}} } \right)^{2} } } }} \hfill \\   {\text{RMSE}} = \sqrt {\frac{1}{N}\sum\limits_{{i = 1}}^{N} {\left( {Y_{{{\text{pred}}}}  - Y_{{{\text{act}}}} } \right)^{2} } }  \hfill \\   {\text{MAE}} = \frac{1}{N}\sum {\left( {Y_{{{\text{pred}}}}  - Y_{{{\text{act}}}} } \right)}  \hfill \\   a{\text{SD}} = \sqrt {\frac{1}{{N - 1}}\sum\limits_{{i = 1}}^{N} {\left( {\left( {a*y_{{{\text{pred}}}}  + b} \right) - y_{{{\text{act}}}} } \right)^{2} } }  \hfill \\   {\text{CI}} = \frac{1}{Z}\sum\limits_{{y_{{act_{i} }}  > y_{{act_{j} }} }} {h\left( {y_{{{\text{pred}}_{i} }}  - y_{{{\text{pred}}_{j} }} } \right)}  \hfill \\  \end{gathered}   $$

where

*N*: the number of protein-ligand complexes

$Y_{\text {act}}$: experimentally measured actual binding affinity values for the protein-ligand complexes

$Y_{\text {pred}}$: the predicted binding affinity values for the given protein-ligand complexes

$y_{\text {act}_i}$ and $y_{\text {pred}_i}$: respectively the actual and predicted binding affinity value of the $i^{th}$ protein-ligand complex

*a*: is slope

*b*: interpretation of the linear regression line of the predicted and actual values. *Z*: the normalization constant, i.e. the number of data pairs with different label values.

*h*(*u*): the step function that returns 1.0, 0.5, and 0.0for $u>0$, $u = 0$, and $u<0$ respectively.

## Data Availability

The program and corresponding data are publicly available on the website https://gitlab.com/mahnewton/daap.
